# List of macrobenthic species: Data from the siberian seas and the adjacent area of the deep-sea central arctic

**DOI:** 10.1016/j.dib.2021.107115

**Published:** 2021-05-12

**Authors:** A.A. Vedenin, S.V. Galkin, A.V. Gebruk

**Affiliations:** Shirshov Institute of Oceanology, Russian Academy of Sciences

**Keywords:** Macrobenthos, Artic ocean, List of species, Benthic diversity

## Abstract

An annotated species list of all macrobenthic invertebrates inhabiting the Siberian sector of the Arctic Ocean is presented. The area considered includes the Kara, Laptev and East Siberian seas and the adjacent region of the deep-sea Central Arctic. Entries on species occurrences in the database are supported by corresponding references. Species of Polychaeta, Crustacea and Echinodermata in addition contain information on bathymetric distribution. Apart from published data, 12 taxa were identified in the area for the first time. In total 1574 macrobenthic species were recorded within the considered area. The most species rich was the Kara Sea with 1184 species. The Laptev and East Siberian seas and the Central Arctic showed lower species richness with correspondingly 1105, 780 and 268 species. The much smaller numbers of species in the East Siberian Sea and in the deep-sea Central Arctic can be related to taxonomic impoverishment or/and much smaller study effort in those regions.

## Specifications Table

**Table:** 

Subject	Earth and Planetary Sciences
Specific subject area	List of macrobenthic species in the Siberian area of the Arctic Ocean, with notes about horizontal and vertical distribution.
Type of data	Tables Graph Figure
How data were acquired	Literature analysis; identification of macrobenthic species in samples obtained in expeditions of the P.P. Shirshov Institute of Oceanology (IORAS); Ocean Data View and Mircosoft Office software.
Data format	Raw Analysed
Parameters for data collection	Analysis of literature; Species identification from benthic samples
Description of data collection	Data were collected by identifying macrobenthic species from samples obtained in expeditions by the Shirshov Institute of Oceanology. Analysis of previously published investigations about macrobenthic species distribution within the study area (the Kara, Laptev and East Siberian seas, and the adjacent area of the deep-sea Central Arctic).
Data source location	Institution: Shirshov Institute of Oceanology, Russian Academy of Sciences Moscow, Russia Data collected from the area of the Kara, Laptev and East Siberian seas, and the adjacent area of the deep-sea Central Arctic. Details on data sources are listed in Table 1 and in Mendeley Data repository, DOI: 10.17632/8fmmdgj8pn.1
Data accessibility	Repository name: Mendeley Data [1] Data identification number: http://dx.doi.org/10.17632/8fmmdgj8pn.1

## Value of the Data

• The list of macrobenthic species occurring within a large area of the Siberian Arctic sector is presented for the first time since 2001. Over almost 20 years since this publication, many new species were described from the region and revisions of various taxa were published; those revisions were followed for the correct synonymy.
• The distribution of three macrotaxa (Polychaeta, Crustacea and Echinodermata) was analysed in details, including the bathymetric range. This is the first summarized data on the upper-most and deeper-most findings of every species of Polychaeta, Crustacea and Echinodermata within the Central Arctic and Siberian Seas. Thirty-nine new species were added to the total species list based on original examined samples. This will be important for any future research on the Arctic biodiversity.
• The data will be useful for future studies about Arctic Ocean biogeography and for different investigations about the Arctic environmental state and, possibly, Climate changing.

## 1 Data Description

**Fig. 1:**
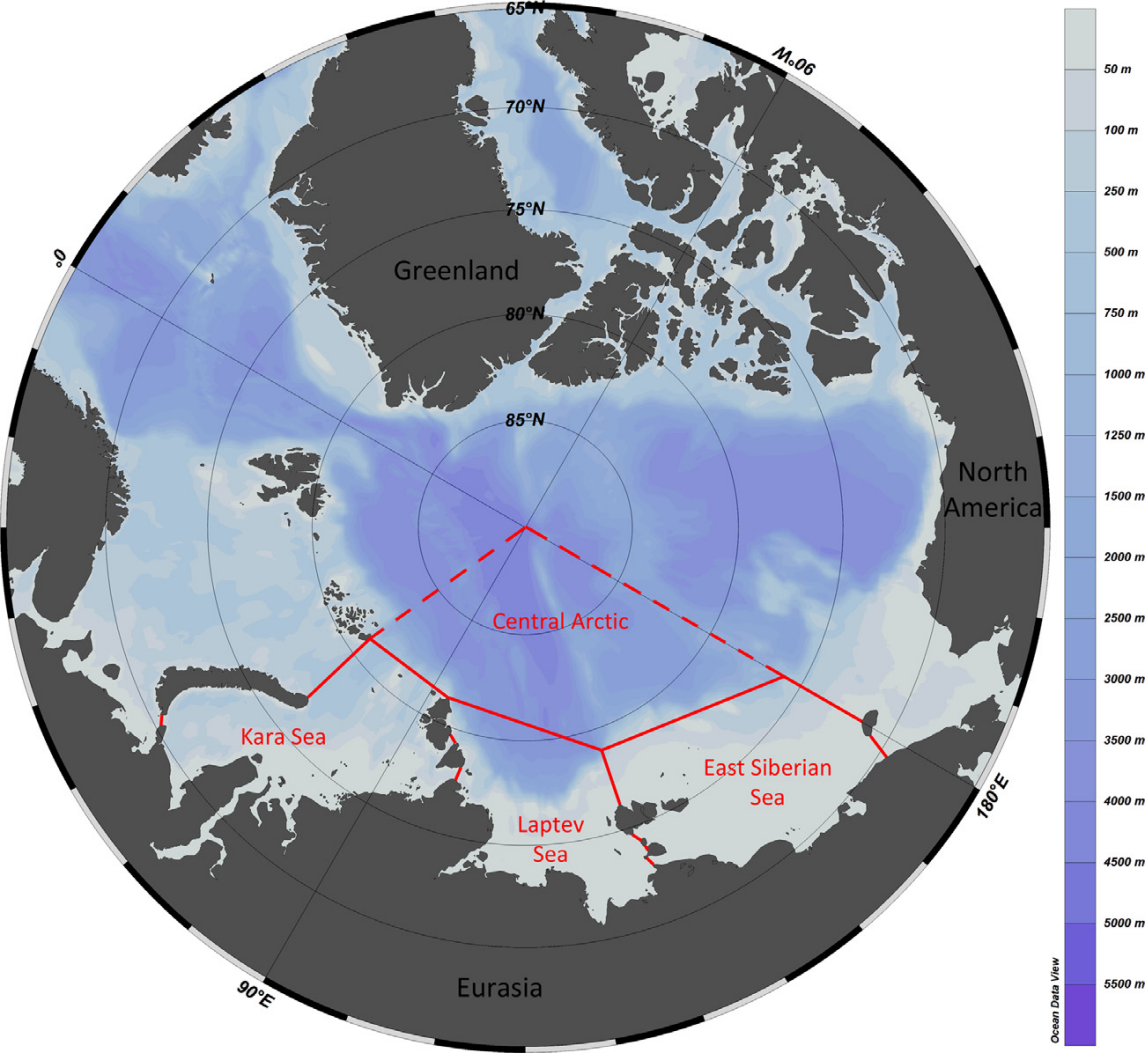
Study area with formal borders of the Kara, Laptev and East Siberian seas. The adjacent sector of the Central Arctic Basin is outlined with dashed lines - the uniformity of fauna of the entire deep-sea Central Arctic is suggested.

**Fig. 2:**
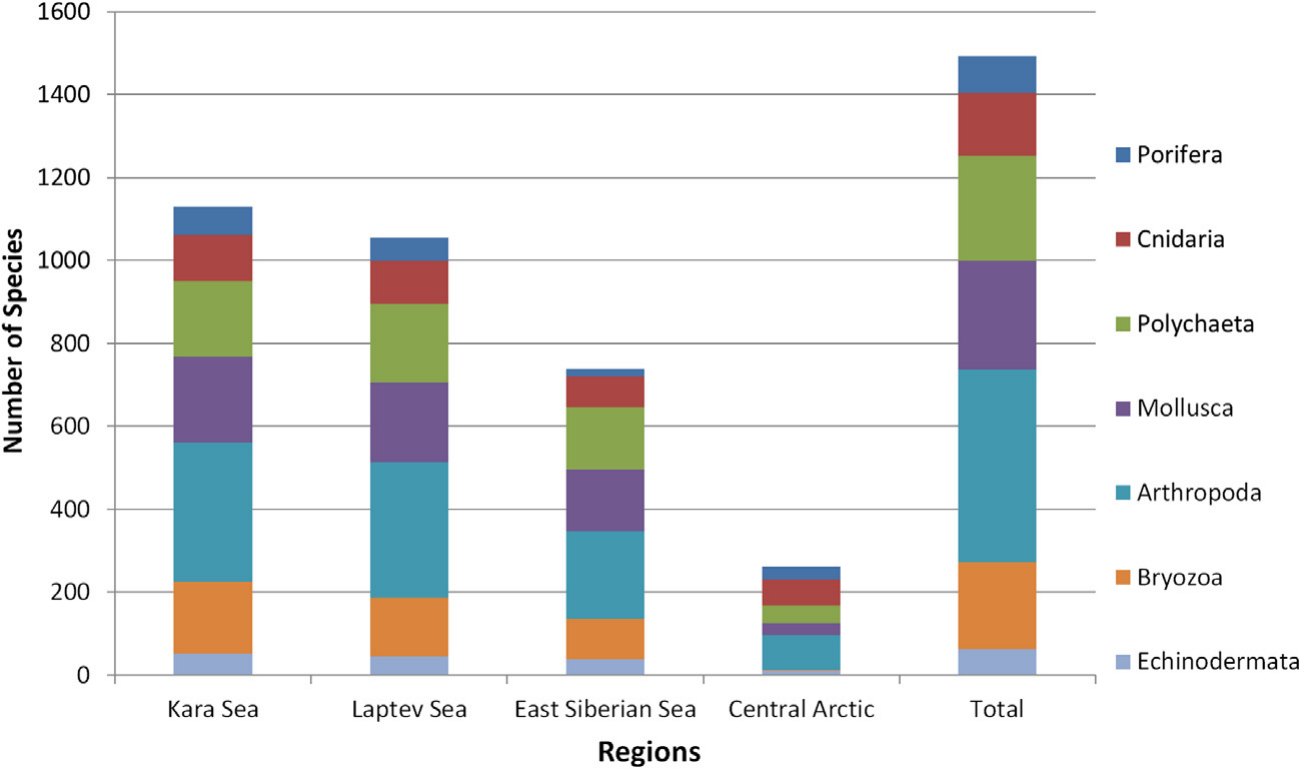
The contribution of major macrotaxa to the species number per basin.

Data reported in the present study are based on the detailed analysis of published information on macrobenthic species occurring in the Kara, Laptev and East Siberian Seas and the adjacent sector of the Central Arctic Basin (Fig. 1). The complete species list is presented in Mendeley Data [1]. In addition to published records, new unpublished occurrences are given in [1]. Polychaeta, Crustacea and Echinodermata are supplemented by information on the shallowest and the deepest findings (the depth range within the Siberian Arctic sector). All unpublished records are listed in Table 1 with information on the year and name of expedition, station number, coordinates and depth. The total number of species per macrotaxa within each basin and the total species number per basin are shown in Table 2. Contribution of the most diverse macrotaxa (Porifera, Cnidaria, Polychaeta, Mollusca, Arthropoda, Bryozoa and Echinodermata) to the species number is shown in Fig. 2.

**Table 1:** List of stations with the expedition number, year, coordinates, depth and gear name for all the unpublished samplings listed in [1].

Station	Expedition	Year	Latitude (N)	Longitude (E)	Depth (m)	Gear
2186	ARK-VIII/3	1991	88.5120	139.9068	1867	Box-corer
2213	ARK-VIII/3	1991	80.4730	8.2052	888	Box-corer
4983	AMK-54	2007	76.9200	70.2700	555	Sigsbee trawl
4985	AMK-54	2007	76.7833	70.6167	465	Sigsbee trawl
4987	AMK-54	2007	76.6583	71.0483	275	Sigsbee trawl
4988	AMK-54	2007	76.5883	71.2567	160	Sigsbee trawl
5002	AMK-54	2007	75.1645	72.6087	26	Sigsbee trawl
5020	AMK-59	2011	73.7176	79.3896	29	Sigsbee trawl
5024	AMK-59	2011	74.9486	77.9020	34	Sigsbee trawl
5026	AMK-59	2011	75.9970	76.6741	63	Sigsbee trawl
5033	AMK-59	2011	77.2100	78.1277	120	Sigsbee trawl
5034	AMK-59	2011	77.4263	77.5674	220	Sigsbee trawl
5039	AMK-59	2011	78.0074	74.8968	364	Sigsbee trawl
5042	AMK-59	2011	78.4915	72.8047	472	Sigsbee trawl
5051	AMK-59	2011	75.8272	68.9850	351	Sigsbee trawl
5054	AMK-59	2011	72.9301	58.3447	376	Sigsbee trawl
5220	AMK-63	2015	73.3255	130.4883	24	Sigsbee trawl
5222	AMK-63	2015	75.8018	130.4882	49	Sigsbee trawl
5225	AMK-63	2015	78.3747	130.6585	2390	Sigsbee trawl
5239	AMK-63	2015	78.5908	88.0673	230	Sigsbee trawl
5258	AMK-63	2015	72.5397	55.5013	34	Okean grab
5263	AMK-63	2015	71.9238	55.3913	18	Sigsbee trawl
AMK-63 C-1	AMK-63	2015	72.5587	55.3535	0	By hand
AMK-63 A-1	AMK-63	2015	71.9396	55.3127	2	Small ichtyological trawl
AMK-63 A-3	AMK-63	2015	71.9287	55.2979	6	Small ichtyological trawl
5590/2	AMK-69	2017	77.1668	114.6804	60	Sigsbee trawl
5635	AMK-69	2017	78.0386	115.8382	777	Sigsbee trawl
5963	AMK-72	2018	78.1801	116.6387	1472	Sigsbee trawl
125–30	PSh-125	2013	76.3533	88.8250	47	Sigsbee trawl
125–32	PSh-125	2013	77.1187	87.6292	149	Sigsbee trawl
125–34	PSh-125	2013	78.0150	87.6317	108	Sigsbee trawl
128–44	PSh-128	2014	79.3208	73.1127	472	Sigsbee trawl
128–45	PSh-128	2014	76.6443	71.0895	273	Sigsbee trawl
128-B1	PSh-128	2014	75.6766	63.6531	0	By hand
128-Ts4	PSh-128	2014	74.3730	58.6116	2	Small ichtyological trawl
128–63 C4	PSh-128	2014	72.5615	55.4210	3	Small ichtyological trawl
128–66	PSh-128	2014	71.9363	55.3319	15	Okean grab
PS80/205–1	ARK-XXVII/3	2012	81.4802	31.0252	615	Agassiz-trawl
PS80/290–3	ARK-XXVII/3	2012	79.6643	130.5948	3398	Amphipod-trap
PS80/334–1	ARK-XXVII/3	2012	85.1632	123.0003	4356	Amphipod-trap
PS80/371–1	ARK-XXVII/3	2012	88.7628	55.6732	4369	Amphipod-trap
SV-IV	ARK-XXX-1	2016	79.1308	4.9063	1540	Box-corer
HG-VI	ARK-XXX-1	2016	79.0562	3.5987	3356	Box-corer

ARK – RV “Polarstern”; AMK – RV “Akademik Mstislav Keldysh”; Psh – RV “Professor Shtokman”.

**Table 2:** Number of species for each of the macrotaxa recorded in Kara, Laptev, East Siberian seas and adjacent area of the Central Arctic basin.

Macrotaxon	Kara Sea	Laptev Sea	East Siberian Sea	Central Arctic	Total
Porifera	69	56	18	31	88
Cnidaria	112	103	75	62	152
Polychaeta	181	191	150	43	253
Oligochaeta	1	1	7	1	9
Hirudinea	5	2	2	0	5
Echiura	2	3	0	2	3
Sipuncula	7	6	5	3	7
Plathelminthes	0	3	3	0	6
Nemertea	7	3	3	0	7
Mollusca	208	191	149	29	263
Arthropoda	334	328	210	84	464
Priapulida	3	3	3	0	3
Brachiopoda	2	1	2	0	2
Bryozoa	173	141	98	2	209
Echinodermata	53	45	39	10	63
Hemichordata	2	1	0	1	4
Chordata (Tunicata)	25	27	16	0	36
**SUM**	1184	1105	780	268	1574

## 2 Experimental Design, Materials and Methods

Primary data were taken from tables published by [2] listing invertebrate species known by that time from the Kara, Laptev and East-Siberian seas and the adjacent Central Arctic Ocean (Fig. 1). We built our new data set on [2] having updated it based on the species lists for the Laptev and East Siberian seas published later [3], [4]. In addition, we extracted relevant information (such as descriptions of new species) from a number of taxonomic revisions of various taxa (such as Porifera, Cnidaria, Polychaeta, Oligochaeta, Crustacea, Pantopoda, Bryozoa, Echinodermata and Hemichordata). We didn't include in our data set the information from [2] on the protozoan, pelagic and meiobenthic taxa such as Foraminifera, Kinorhyncha, Rotatoria, Copepoda and Nematoda and some Cnidaria (lacking benthic stage). The area marked as “the Central Arctic” in Fig. 1 is bordered by dashed lines since we also considered species recorded from adjacent areas owing to supposed uniformity of fauna of central Arctic basins [5]. Thus, we listed under “Central Arctic” some records e.g. from the western part of the Nansen Basin [1], [6].

For Polychaeta, Mollusca and Echinodermata we analysed published information to establish the maximum species depth range within our study area, with the shallowest and the deepest known records. The corresponding references are shown in [1]. A number of taxa were identified based on samples obtained by the IORAS expeditions; this is original not published earlier information (Table 1). Some of those taxa were new to science, whereas for others the known depth or geographic ranges were extended (in [1], marked as “our unpublished data”; Table 1). In total 12 species were identified not recorded before in the area under consideration. The station numbers for each of these taxa are reported in Table 1. The information about the depth, coordinates, sampling gear, and expedition of corresponding stations is shown in Table 1. Species names were verified according to the World Register of Marine Species (WoRMS, http://marinespecies.org/).

Overall 1574 species of macrobenthos were identified from the area of the Kara, Laptev and East Siberian seas and the adjacent part of the deep-sea Central Arctic (Table 2). The most species rich appeared the Kara Sea with 1184 species. The Laptev and East Siberian seas showed lower species richness with 1105 and 780 species correspondingly. In the deep-sea Central Arctic the value was the lowest - only 268 species (Table 2). The contribution of major macrotaxa to the species number per basin is shown in Fig. 2. The results for the East Siberian Sea and for the Central Arctic can be related not only to the true taxonomic impoverishment, but also to the overall diversity underestimation owing to much smaller general sampling effort in these basins.

## Ethics Statement

This work didn't involve human or animal experiments. All data were taken from the previously sampled collections or previously published studies.

## CRediT Author Statement

**Vedenin AA:** Conceptualization, Methodology, Software, Investigation, Original draft preparation; **Galkin SV:** Data curtion; **Gebruk AV:** Supervision, Validation, Reviewing and Editing.

## Declaration of Competing Interest

The authors declare that they have no known competing financial interests or personal relationships.
